# Selective hydrogenation of 5-(hydroxymethyl)furfural to 5-methylfurfural over single atomic metals anchored on Nb_2_O_5_

**DOI:** 10.1038/s41467-020-20878-7

**Published:** 2021-01-26

**Authors:** Shaopeng Li, Minghua Dong, Junjuan Yang, Xiaomeng Cheng, Xiaojun Shen, Shulin Liu, Zhi-Qiang Wang, Xue-Qing Gong, Huizhen Liu, Buxing Han

**Affiliations:** 1 Beijing National Laboratory for Molecular Sciences, CAS Key Laboratory of Colloid and Interface and Thermodynamics, Institute of Chemistry, Chinese Academy of Sciences, 100190 Beijing, China; 2 School of Chemistry and Chemical Engineering, University of Chinese Academy of Sciences, 100190 Beijing, China; 3 Key Laboratory for Advanced Materials, Centre for Computational Chemistry and Research Institute of Industrial Catalysis, School of Chemistry and Molecular Engineering, East China University of Science and Technology, 200237 Shanghai, China; 4 Physical Science Laboratory, Huairou National Comprehensive Science Center, 101407 Beijing, China

**Keywords:** Heterogeneous catalysis, Sustainability, Density functional theory

## Abstract

5-Methylfurfural (MF) is a very useful chemical. Selective hydrogenation of biomass platform molecule 5-(hydroxymethyl)furfural (HMF) to MF using H_2_ as the reducing agent is very attractive, but challenging because hydrogenation of C=O bond in HMF is more favourable than C–OH both kinetically and thermodynamically, and this route has not been realized. In this work, we prepare isolated single atomic catalysts (SACs) Pt_1_/Nb_2_O_5_-Ov, Pd_1_/Nb_2_O_5_-Ov, and Au_1_/Nb_2_O_5_-Ov, in which single metal atoms are supported on oxygen defective Nb_2_O_5_ (Nb_2_O_5_-Ov). It is discovered that the SACs can efficiently catalyze the hydrogenation of HMF to MF using H_2_ as the reducing agent with MF selectivity of >99% at complete conversion, while the selectivities of the metal nanocatalysts supported on Nb_2_O_5_ are very poor. A combination of experimental and density function theory (DFT) studies show that the unique features of the SACs for the reaction result from the cooperation of the Nb and Pt sites near the interface in the Pt_1_/Nb_2_O_5_-Ov. The Pt atoms are responsible for the activation of H_2_ and the Nb sites activate C-OH in the reaction. This work opens the way for producing MF by direct hydrogenation of biomass-derived HMF using H_2_ as the reductant.

## Abstract (web summary)

Selective hydrogenation of 5-(hydroxymethyl)furfural (HMF) to 5-Methylfurfural using H2 as reductant is very attractive, but remains challenging. Here, the authors report that isolated single atomic catalysts can catalyze the reaction efficiently with selectivity >99% at complete conversion of HMF.

## Introduction

Selective hydrogenation is a critical class of reactions, and selectivity is an essential parameter in a chemical reaction. There are often different kinds of unsaturated groups in a compound such as C=C, C=O, C≡C, C=N, aromatic ring, and -NO_2_. Exploration of the methods to reduce one or some functional groups selectively while retaining others not only can synthesize many value-added chemicals but also can broaden chemical knowledge.

5-(Hydroxymethyl)furfural (HMF) is an important biomass platform compound and it can be produced from cellulose that covers about 40% of lignocellulosic biomass^1,2^. The reduction of HMF is a very promising route to produce high-value chemicals. It has been reported that various products such as 2,5-bis(hydroxymethyl)furan (DHMF), 2,5-dimethylfuran (DMF), 2,5-dimethyltetrahydrofuran (DMTHF), 2,5-hexanediole (HDO) could be produced by the selective hydrogenation of HMF^3–10^. 5-Methylfurfural (MF) is a very useful chemical, which can be used as a food additive and commonly used synthetic intermediate^11–14^. However, the selective hydrogenation of the C–OH group in HMF to 5-methylfurfural (MF) has not been realized using H_2_ as the reducing agent, since in general the C=O bond is easier reduced than the C–OH bond in the hydrogenation of HMF from both kinetic and thermodynamic aspects^15^. Kinetically, hydrogenation of C=O bond in HMF is more favorable than the C–OH. Thermodynamically, MF has a strong tendency to be further hydrogenated to DMF. Ankur Bordoloi et al. studied the three reducible bonds in HMF (C=O, C=C, and C–OH) and found that C=O is the most prone to hydrogenation because of the strongest electrophilicity of aldehyde carbon. Furthermore, the activation energy required by the hydrogenation of C=O is much lower than that required by the dehydroxylation of C–OH^16^.

**Fig. 1:**
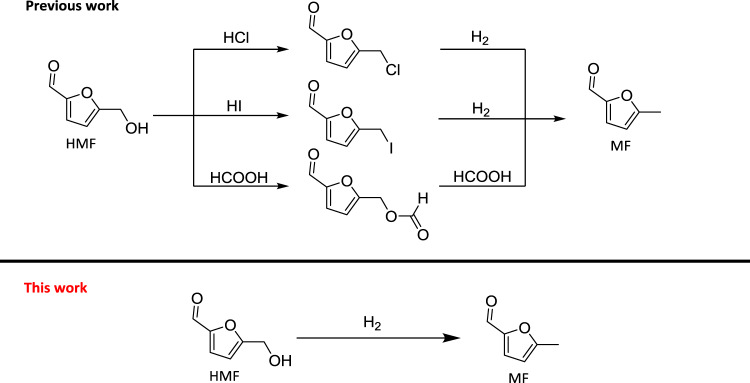
The synthetic routes to produce MF from HMF. The previous methods to synthesize MF with acid. This work to directly synthesize MF without any additives.

Synthesis of MF from HMF was reported by indirect routes, which involved in the transformation of HMF to halogen compounds, such as 5-chloromethylfurfural (CMF) or 5-iodomethylfurfural (IMF), and MF was then prepared by the hydrodehalogenation of CMF or IMF (Fig. 1)^17–19^. The selective hydrogenolysis of HMF to MF was also reported using HCOOH as the reducing agent and the process proceeded via an esterification procedure. Unfortunately, the author found that no MF was detected when H_2_ was used^20^. Obviously, it is very attractive to prepare MF directly from the selective hydrogenation of HMF using H_2_ as the reducing agent because the process is simple, and the only byproduct is H_2_O.

Design of ideal catalysts is the key to achieve the selective hydrogenation of HMF to MF. In recent years, isolated single atomic catalysts (SACs) have been used in many reactions. For example, Pt_1_/N-C SACs showed high chemo- and regioselectivity towards terminal alkynes in hydrogenation^21^. An atomically dispersed copper (Cu) catalyst supported on defective nanodiamond graphene exhibited excellent catalytic performance for the selective conversion of acetylene to ethylene^22^. The Pt_1_/α-MoC catalyst showed promising activity and a strong chemospecificity towards the hydrogenation of nitro groups^23^. SACs have also been used to catalyze other important reactions, such as selective hydrogenation of nitroarenes, alkenes and carbonyl compounds, the catalytic transformation of methane, aqueous reforming of methanol, hydroformylation of olefins, olefin metathesis, and oxygen reduction, in which they showed excellent performance^24–31^.

Nb_2_O_5_ has been used in the activation and cleavage of C_aliphatic_–O and C_aromatic_–O bonds in both lignin and its model compounds^32–34^. In this work, we fabricated Pt_1_/Nb_2_O_5_-Ov, Pd_1_/Nb_2_O_5_-Ov, and Au_1_/Nb_2_O_5_-Ov catalysts, in which single atomic metal sites were supported on oxygen defective Nb_2_O_5_ (Nb_2_O_5_-Ov). Very interestingly, it was discovered that they could efficiently catalyze selective hydrogenation of HMF to MF with >99% selectivity at complete conversion. The density function theory (DFT) calculations and experimental results indicated that the unusual feature of the catalysts for the reaction resulted from the excellent cooperation of the Pt and Nb sites. The Pt sites were responsible for the activation of H_2_ and the Nb sites near Pt sites selectively activated the C–OH group in HMF, and thus very high selectivity was achieved.

## Results

### Structural characterizations

**Fig. 2:**
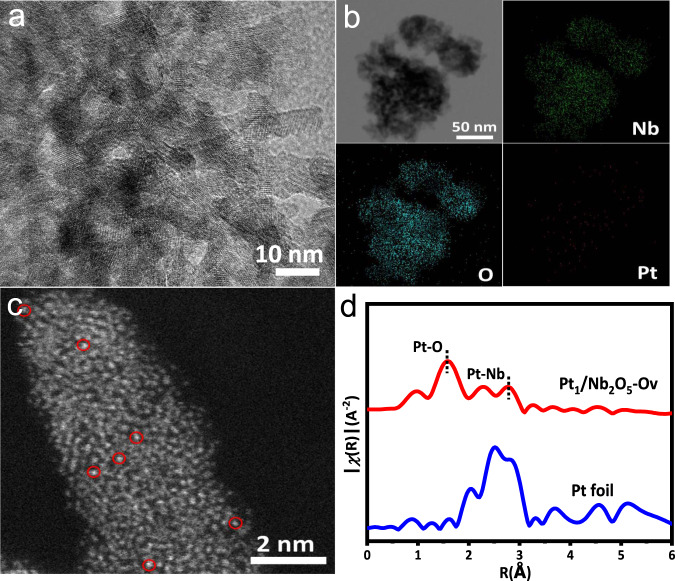
The structural characterization of Pt_1_/Nb_2_O_5_-Ov. **a** TEM images of Pt_1_/Nb_2_O_5_-Ov. **b** EDS mapping images of Pt_1_/Nb_2_O_5_-Ov. **c** AC–HAADF–STEM image of Pt_1_/Nb_2_O_5_-Ov. The atomically dispersed Pt atoms are highlighted by red circles. **d** The *k*^2^-weighted Fourier transform spectra of Pt_1_/Nb_2_O_5_-Ov and Pt foil.

The Nb_2_O_5_ with oxygen-vacancy defects (Nb_2_O_5_-Ov) were prepared by thermal treating Nb_2_O_5_ in reducing atmosphere for 4 h at 500 °C (reducing gas contains 10 vol.% hydrogen and 90 vol.% argon). Defects of oxygen vacancies were detected by electron-paramagnetic resonance (EPR) measurement (Supplementary Fig. 1). A signal of oxygen vacancy at a *g* value of 2.003 was observed for Nb_2_O_5_-Ov and Pt_1_/Nb_2_O_5_-Ov, while no detectable EPR signal was observed for the intrinsic Nb_2_O_5_. In addition, the oxygen vacancy concentration of Pt_1_/Nb_2_O_5_-Ov was the highest. The transmission electron microscopy (TEM) image of the prepared Pt_1_/Nb_2_O_5_-Ov is shown in Fig. 2a. No Pt cluster or particle can be observed on the surface of Nb_2_O_5_-Ov, while the mapping images suggest the uniform distributions of Nb, O, and Pt over the entire architecture (Fig. 2b). The aberration-correction high-angle annular dark-field scanning transmission electron microscopy (AC–HAADF–STEM) image exhibited some marked bright points on the surface of Nb_2_O_5_-Ov, indicating the atomically dispersed Pt on the defective support (Fig. 2c). The content of Pt in Pt_1_/Nb_2_O_5_-Ov was 0.039 wt%, which was analyzed by inductively coupled plasma (ICP) mass spectrometry. Fourier-transformed *k*^2^-weighted extended X-ray absorption fine structure (EXAFS) in R space was performed to elucidate the coordination environments of Pt atoms anchored on Nb_2_O_5_-Ov. It was shown that there was only one notable peak at 1.5 Å from the Pt–O contribution, and no peak at 2.5 Å from the Pt-Pt contribution, confirming the sole presence of dispersed single Pt atoms in Pt_1_/Nb_2_O_5_-Ov (Fig. 2d). The least-squares EXAFS fitting curves of Pt_1_/Nb_2_O_5_-Ov and Pt foil and PtO_2_ are shown in Supplementary Fig. 2, and the corresponding structure parameters are listed in Supplementary Table 1.

**Fig. 3:**
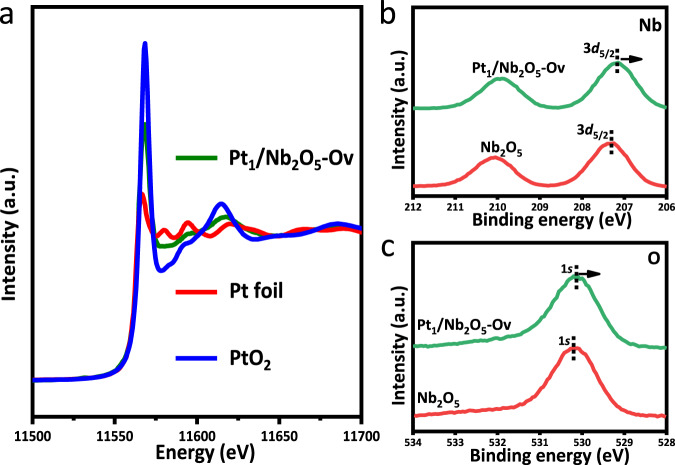
The electron state characterization of Pt_1_/Nb_2_O_5_-Ov. **a** The XANES spectra at the Pt L3-edge. **b** The XPS spectra of Nb 3*d* and **c** O1*s* of Pt_1_/Nb_2_O_5_-Ov and Nb_2_O_5_.

Pt species in Pt_1_/Nb_2_O_5_-Ov were partially positively charged as evidenced by X-ray absorption near-edge structure (XANES) of Pt_1_/Nb_2_O_5_-Ov sample between Pt foil and PtO_2_ (Fig. 3a). X-ray photoelectron spectroscopy (XPS) was used to study the valence states of Nb and O. As shown in Fig. 3b, Nb 3*d*_5/2_ binding energy of intrinsic Nb_2_O_5_ was 207.3 eV, which shifted to 207.1 eV for Pt_1_/Nb_2_O_5_-Ov. O 1*s* binding energy of intrinsic Nb_2_O_5_ was 530.3 eV, which shifted to 530.1 eV for Pt_1_/Nb_2_O_5_-Ov (Fig. 3c). The Pt 4*f* peak for the Pt_1_/Nb_2_O_5_-Ov sample can be deconvoluted into two peaks (Supplementary Fig. 3). The peaks at 76.1 and 72.9 eV correspond to the Pt^δ+^4*f*_5/2_ and Pt^δ+^4*f*_7/2_, respectively^35–37^. These results imply that there is a strong electron interaction between Nb_2_O_5_-Ov and Pt, and the electron transfers from Pt to the Nb_2_O_5_-Ov support.

### Catalytic performance for HMF hydrogenation

The performance of Pt_1_/Nb_2_O_5_-Ov catalytic systems was studied for the selective hydrogenation of HMF and the results are shown in Table 1. The reaction could not occur in the absence of any catalyst (Table 1, entry 1). Very interestingly, Pt_1_/Nb_2_O_5_-Ov could efficiently catalyze the selective hydrogenation of HMF to MF with >99% selectivity at complete conversion of HMF in 4 h. The turnover frequency of producing MF could reach 1875 h^−1^ (Table 1, entry 2). The effects of different reaction temperatures and H_2_ pressures on the performance of Pt_1_/Nb_2_O_5_-Ov catalysts were studied. When the temperature changes from 160 °C to 120 °C, H_2_ pressure changes from 4.0 MPa to 1.0 MPa, the selectivities to MF are always >99% (Table 1, entry 3–6). These results indicate that the selectivity of MF was independent of H_2_ pressure and reaction temperature. The catalytic performances of Pt-based catalysts for the conversion of HMF are summarized in Supplementary Table 2.

**Table 1:** Selective hydrogenation of HMF over Pt_1_/Nb_2_O_5_-Ov catalysts^[a]^.


Entry	Catalyst	Conversion (%)	Selectivity^c^ (%)				TOF/h^b^
			1	2	3	4	
1	–	–	–	–	–	–	0
2	Pt_1_/Nb_2_O_5_-Ov	>99	>99	–	No	–	1875
3^d^	Pt_1_/Nb_2_O_5_-Ov	92	>99	–	No	–	1742
4^e^	Pt_1_/Nb_2_O_5_-Ov	86	>99	–	No	–	1628
5^f^	Pt_1_/Nb_2_O_5_-Ov	71	>99	–	No	–	1344
6^g^	Pt_1_/Nb_2_O_5_-Ov	35	>99	–	No	–	656

^a^Reaction conditions: HMF (0.3 mmol), catalyst (20 mg), solvent (THF 2 mL), reaction temperature (160 °C), H_2_ pressure (4.0 MPa), reaction time (4 h), stirring speed (600 rpm).

^b^TOF = moles of product × moles of metal^−1^ × h^−1^.

^c^The selectivity was calculated based on the product detected by gas chromatography.

^d^Reaction temperature (160 °C), H_2_ pressure (2.0 MPa).

^e^Reaction temperature (160 °C), H_2_ pressure (1.0 MPa).

^f^Reaction temperature (140 °C), H_2_ pressure (4.0 MPa).

^g^Reaction temperature (120 °C), H_2_ pressure (4.0 MPa).

**Fig. 4:**
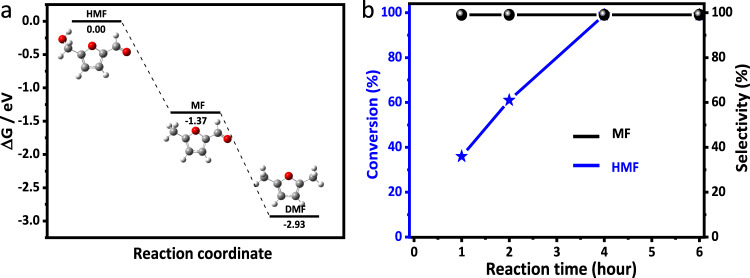
Factors affected the selective hydrogenation of HMF to MF. **a** Gibbs free energy of HMF hydrogenation to MF and DMF from DFT calculation. C, H, and O atoms are represented as silver, white, and red balls. **b** The effect of reaction time over Pt_1_/Nb_2_O_5_-Ov.

The quantum chemical calculations demonstrated that HMF could be hydrogenated to produce MF and DMF with Gibbs free energy ($\Delta$G) of −1.37 and −2.93 eV, respectively (Fig. 4a and Supplementary Table 3), indicating that MF is a thermodynamically unstable compound in the reaction. Even if MF is produced from the hydrogenation of HMF, it has the thermodynamic potential to be further hydrogenated to DMF. The variation of conversion and selectivity with reaction time over Pt_1_/Nb_2_O_5_-Ov was studied and the results are shown in Fig. 4b. It was found that the selectivity of MF was independent of the conversion of HMF and could reach >99% of all the reaction times. The conversion of HMF approached 100% at 4 h. Moreover, even the reaction time was prolonged to 6 h, the selectivity of MF was still >99%.

## Discussion

To unveil the reason for the high MF selectivity of Pt_1_/Nb_2_O_5_-Ov in selective hydrogenation of HMF, the selective chemisorption of C–OH and C=O on the surface of Pt_1_/Nb_2_O_5_-Ov was investigated by FT-IR spectroscopy using methanol and n-propanal as model molecules. It was found that methanol dissociates into methoxy species on Pt_1_/Nb_2_O_5_-Ov, and the bands of the methoxy species observed at 1218 cm^−1^ and 1155 cm^−1^ can be assigned to the ν(C–O) bands of the on-top and bridged sites, respectively (Supplementary Fig. 4). This is the direct evidence of chemical adsorption C–OH by Pt_1_/Nb_2_O_5_-Ov. However, Pt_1_/Nb_2_O_5_-Ov could not adsorb C=O groups chemically. The FT-IR spectroscopy experimental results indicate that C–OH in HMF is selectively activated over Pt_1_/Nb_2_O_5_-Ov. To further prove this, MF was also used as the substrate to check the performance of Pt_1_/Nb_2_O_5_-Ov. MF could not be converted over the Pt_1_/Nb_2_O_5_-Ov (Supplementary Table 4), indicating that it is not active for the hydrogenation of C=O, which is the main reason for the high selectivity of MF. The heterogeneous nature of Pt_1_/Nb_2_O_5_-Ov was evaluated by removing the catalyst after the reaction was conducted for 1 h, and then the reaction was continued for 5 h without the solid catalyst. The product yield was not further increased without Pt_1_/Nb_2_O_5_-Ov (Supplementary Fig. 5a), indicating no leaching of active species to the reaction mixture. The reusability of the catalyst was tested, and the results are shown in Supplementary Fig. 5b, the yield decreased slightly from run 1 to run 4 due to inevitable loss of catalyst in the recovery process. After the 4th run, 17 mg of Pt_1_/Nb_2_O_5_-Ov (85% original amount) was recovered. From run 5 to run 7, 0.25 mmol of the reactant was added, which was 85% of the first 4 runs. The yield of the product in the 5th run was nearly the same as that of the first run. The yields of runs 6 and 7 were slightly reduced because the slight loss of the catalyst in the recovery process. These results suggest that the intrinsic activity of the catalyst did not decrease during the recycle process. The used catalysts were characterized by HRTEM/elemental mapping and XPS. As shown in Supplementary Fig. 6, no Pt clusters or particles were observed on the surface of the used catalysts. The mapping images indicate the uniform distributions of Nb, O, and Pt over the used catalysts. The binding energy of Pt^δ+^4*f*_5/2_ (76.3 eV) and Pt^δ+^4*f*_7/2_ (73.0 eV) for used catalyst was almost the same as that of original Pt_1_/Nb_2_O_5_-Ov (Supplementary Fig. 7), suggesting that the electron state did not change during the reaction process. Moreover, the content of Pt in used Pt_1_/Nb_2_O_5_-Ov was 0.039 wt%, indicating no Pt leaching. These results show the excellent stability of Pt_1_/Nb_2_O_5_-Ov in the reaction systems.

DFT calculations were performed to study the adsorption of HMF on Nb_2_O_5_ and Pt_1_/Nb_2_O_5_-Ov surfaces. All the calculations were conducted using the Vienna Ab initio Simulation Package (VASP), and the calculation details were provided in the experimental section. The three-layer *p*(3 × 3) surface slab for the Nb_2_O_5_(001) surfaces and Pt atoms modified Nb_2_O_5_ surfaces with (Pt_1_/Nb_2_O_5_-Ov) or without (Pt_1_/Nb_2_O_5_) oxygen vacancies were built (Supplementary Fig. 8). The distances of the Nb3-Pt is 3.363 Å in Pt_1_/Nb_2_O_5_-Ov (Supplementary Fig. 8c), which is consistent with the results observed in the experiment (Supplementary Table 1), indicating that the model we constructed is reasonable. It can be found that oxygen vacancies are easily formed on Pt_1_/Nb_2_O_5_, where the corresponding oxygen vacancy formation energy is exothermic 0.07 eV (Supplementary Fig. 9), indicating the Pt_1_/Nb_2_O_5_-Ov surface is stable.

**Fig. 5:**
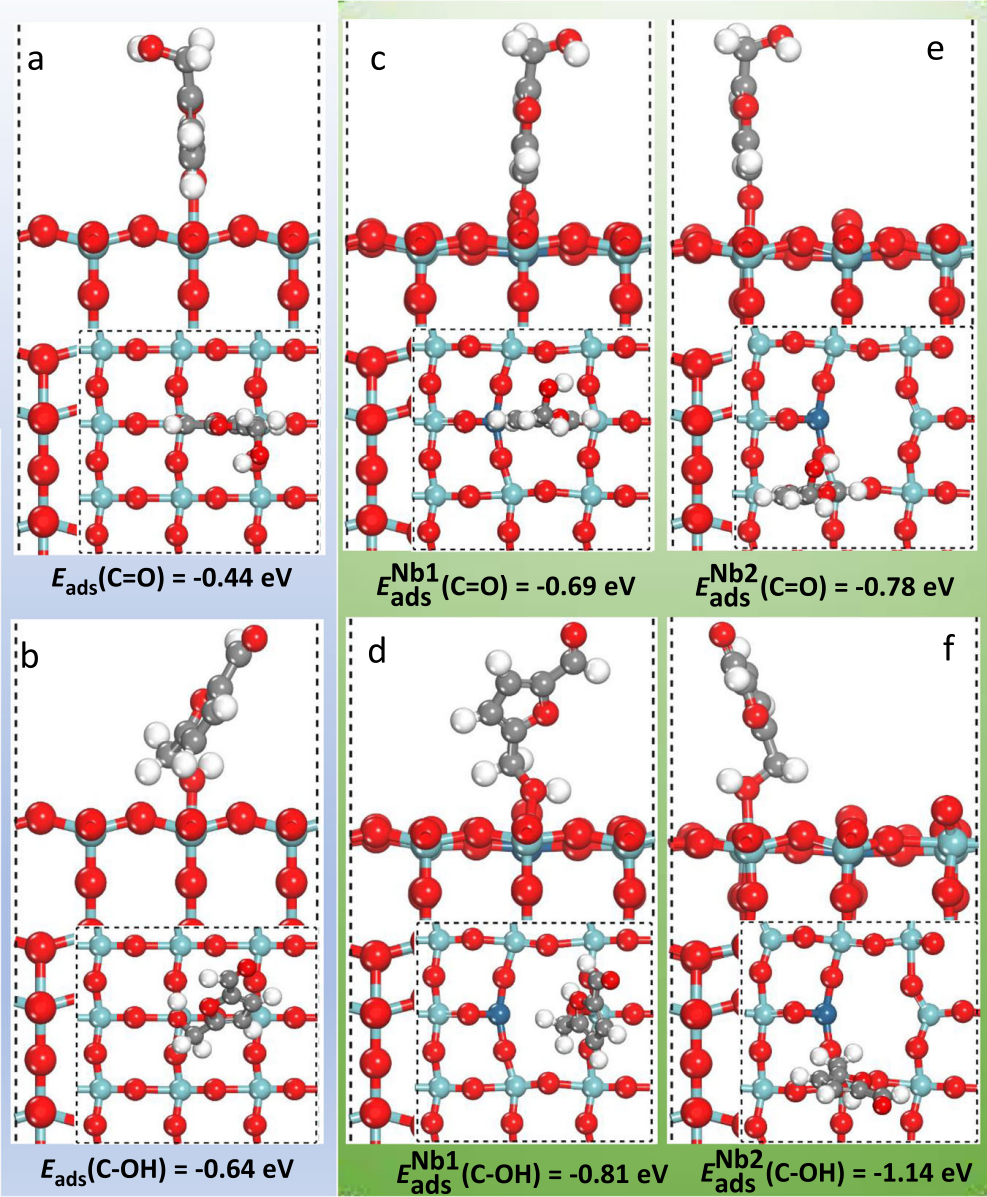
Calculated adsorption energy and structures of HMF. (side view, Inset: top view). **a**, **b** Nb_2_O_5_ surface, which marked with blue background. **c–f** Pt_1_/Nb_2_O_5_-Ov surface, which marked with green background. The adsorption modes for C=O (upper row) and the C–OH (bottom row) in HMF. Silver: C, white: H, red: O, light blue: Nb, blue: Pt.

The adsorption energy of HMF adsorbed on various Nb_2_O_5_ surfaces by C=O adsorption mode or C–OH adsorption mode is first calculated (Fig. 5). The results show that the adsorption energy of HMF by C–OH adsorption mode is higher than the adsorption energy of HMF by C=O adsorption mode on the same sites of Nb_2_O_5_ or Pt_1_/Nb_2_O_5_-Ov surfaces, suggesting the preferential adsorption of C–OH. The adsorption energy of HMF by C–OH adsorption mode on Nb2 site of Pt_1_/Nb_2_O_5_-Ov surfaces was −1.14 eV, which is much higher than the adsorption energy of HMF by C=O adsorption mode (−0.78 eV). Furthermore, Pt and oxygen vacancies promoted the adsorption of HMF as evidenced that the adsorption energy of HMF by C–OH or C=O mode is the highest on Pt_1_/Nb_2_O_5_-Ov surfaces. In addition, we also calculated the H_2_ adsorption on Pt_1_/Nb_2_O_5_-Ov surfaces (Supplementary Fig. 10). The adsorption energy is exothermic 0.51 eV, which is weaker than HMF adsorption.

**Fig. 6:**
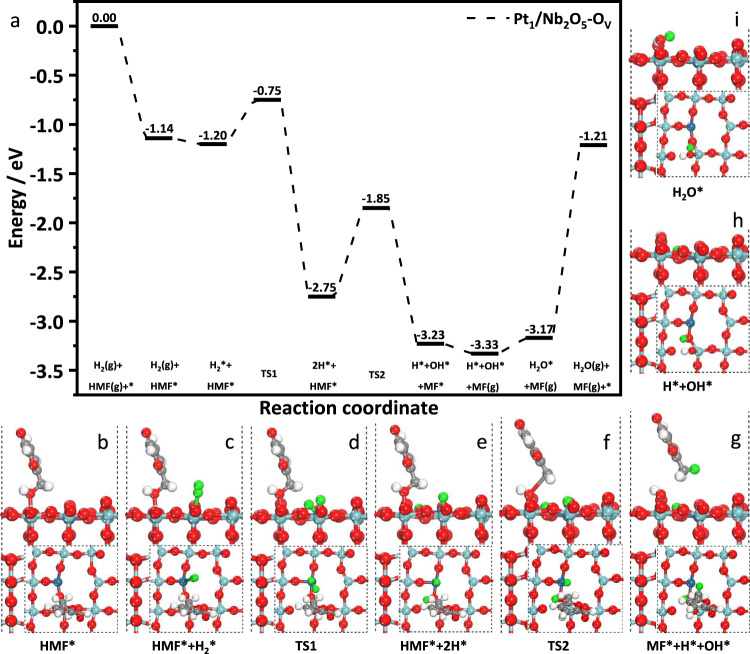
Reaction pathway based on DFT calculations. Calculated energy profile of **a** HMF hydrogenation on Pt_1_/Nb_2_O_5_-Ov surfaces, as well as **b**–**i** are the key structures of HMF hydrogenation. Silver: C, white: H, red: O, light blue: Nb, blue: Pt, green: H (Hydrogen).

It has been reported that the dissociation of H_2_ occurs via a heterolytic pathway over SACs, because all metal atoms are individually dispersed and no metal-metal pairs available for homolytic dissociation of H_2_^38^. The reaction pathway over Pt_1_/Nb_2_O_5_-Ov for the selective hydrogenation of HMF was also calculated by DFT (Fig. 6). First, the C–OH of HMF adsorbed on Nb sites, and then H_2_ adsorbed on Pt was readily split into two H species. One of the H species moved to nearby oxygen on Pt_1_/Nb_2_O_5_-Ov to yield O–H species, leaving the other H on Pt formed Pt–H species. This step was calculated to be exothermic by 1.55 eV and exhibited a barrier of 0.45 eV (from c to e). It is generally accepted that the activity of Pt–H species is higher than that of O–H species^37^. The Pt–H species attacked the C site of C–OH in HMF accompanied by the breakage of C–O, leading to the formation of MF and -OH adsorbed on the Nb site. This step is exothermic 0.48 eV with an energy barrier of 0.90 eV (from e to g). Finally, H_2_O was formed with endothermic 0.16 eV and the entire reaction cycle was completed on Pt_1_/Nb_2_O_5_-Ov surfaces. Therefore, the ability of producing active H species and the adsorption modes of HMF determined the activity and selectivity of HMF hydrogenation reaction on Pt_1_/Nb_2_O_5_-Ov surfaces.

**Fig. 7:**
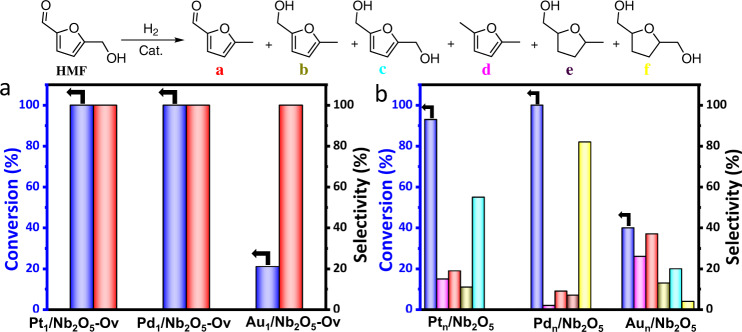
Catalytic performance of M_1_/Nb_2_O_5_-Ov and M_n_/Nb_2_O_5_. Reaction results for the catalyst of **a** Pt_1_/Nb_2_O_5_-Ov, Pd_1_/Nb_2_O_5_-Ov, and Au_1_/Nb_2_O_5_-Ov, reaction time 4 h; **b** Pt_n_/Nb_2_O_5_, Pd_n_/Nb_2_O_5_, and Au_n_/Nb_2_O_5_, reaction time 1 h; reaction condition: substrate (0.3 mmol), catalyst (20 mg), THF (2.0 mL), H_2_ (4.0 MPa), 160 °C. The products were detected by gas chromatography. The black arrows points to the conversion of HMF.

Combining DFT calculation and experimental results, we can conclude that the Pt and Nb sites near the interface in the Pt_1_/Nb_2_O_5_-Ov cooperated very well to promote the selective hydrogenation of HMF to MF. The Pt atom was responsible for the activation of H_2_ and the Nb sites activated C–OH. Following this mechanism, it can be deduced that the selectivity of MF should be independent of the characteristics of metal for SACs. To verify this, Pd_1_/Nb_2_O_5_-Ov and Au_1_/Nb_2_O_5_-Ov were also prepared using the same method with Pt_1_/Nb_2_O_5_-Ov (the characterization results are shown in Supplementary Figs. 11 and 12) and their catalytic performances were checked (Fig. 7). Both Pd_1_/Nb_2_O_5_-Ov and Au_1_/Nb_2_O_5_-Ov showed >99% selectivity of MF, although the activity of Au_1_/Nb_2_O_5_-Ov was lower, showing that the nature of the metal species in the SACs only affected the activity but not the selectivity, which further supports the argument that the metal atom and the Nb sites activated the H_2_ and C–OH respectively in the reaction. For comparison, we also prepared the supported Pt, Pd, and Au nanocatalysts Pt_n_/Nb_2_O_5_, Pd_n_/Nb_2_O_5_, and Au_n_/Nb_2_O_5_, and used to catalyze the reaction. All the nanocatalysts showed very low selectivity to MF because the multiple active sites provided by nanoparticles could activate H_2_ and different chemical bonds in HMF. The results also further showed that the SACs had unique features for the selective hydrogenation of HMF to MF.

The selectivity of different substrates with OH and CHO over Pt_1_/Nb_2_O_5_-Ov were investigated and the results are given in Supplementary Table 5. It was found that 98% yield of 5-methyl-2-thiophenecarboxaldehyde could be achieved for the selective hydrogenolysis of 5-(hydroxymethyl)thiophene-2-carbaldehyde (Supplementary Table 5, entry 1). The reactivity of furfural and furfuryl alcohol were checked, and it was found that Pt_1_/Nb_2_O_5_-Ov was active for the hydrogenolysis of the furfuryl alcohol to 2-methylfuran while inactive for the hydrogenation of furfural to furfuryl alcohol (Supplementary Table 5, entries 2 and 3). The similar results were obtained for the reactivity of 5-methylfurfural and (5-methyl-2-furyl)methanol (Supplementary Table 5, entries 4 and 5). The conversion of tetrahydrofurfuryl alcohol was very low over the catalyst (Supplementary Table 5, entry 6). The reactivity of benzaldehyde was higher than benzyl alcohol. In all, 24% conversion of benzyl alcohol and 55% conversion of benzaldehyde were obtained under the same reaction conditions (Supplementary Table 5, entries 7 and 8). However, the yield of *p*-methyl benzaldehyde could reach 23% for the selective hydrogenolysis of 4-(hydroxymethyl)benzaldehyde (Supplementary Table 5, entry 9). The catalyst could also catalyze the hydrogenolysis of aliphatic alcohols. The conversion of glycerol was 11% and the conversion of 1,2,6-hexanetriol was 26% (Supplementary Table 5, entries 10 and 11). Unfortunately, Pt_1_/Nb_2_O_5_-Ov could not catalyze the selective hydrogenation of aliphatic compounds with OH and CHO to the corresponding aldehyde (Supplementary Table 5, entries 12 and 13).

In summary, SACs (Pt_1_/Nb_2_O_5_-Ov, Pd_1_/Nb_2_O_5_-Ov, and Au_1_/Nb_2_O_5_-Ov) can efficiently catalyze selective hydrogenation of HMF to MF. The selectivity to MF can be as high as >99% at complete conversion. The unusual and unique property of the SACs for the reaction originates from the excellent cooperation of the Nb and Pt sites near the interface in the catalysts. The Pt atom sites can only activate the H_2_, and the Nb sites solely activate C–OH in the reaction, while none of the Nb and Pt sites can activate the C=O group. Thus, the selectivity of MF is exceptionally high. Moreover, the catalysts can be reused at least 7 times without a decrease in the selectivity. We believe that the SACs and the reaction route have a high potential of application in producing MF from biomass-derived HMF by hydrogenation because of the apparent advantages, such as excellent selectivity, and high efficiency and stability, and the reaction process is very straightforward and uses biomass-derived feedstocks. We also believe that the protocol to combine the active sites of metal and support in SACs can also be used to catalyze some other selective hydrogenation reactions in biomass transformation.

## Methods

### Chemicals and materials

Niobium(V) oxalate hydrate (99%, alfa), cetyl-trimethyl-ammonium bromide (CTAB, 99%, Sigma-Aldrich), and H_2_PtCl_6_·6H_2_O (>99.0%) was purchased from Sinopharm Chemical Reagent Co. Ltd. Tetrahydrofuran, 5-hydroxymethylfurfural, 2,5-dimethylfuran, 2,5-bis(hydroxymethyl)furan, 2,5-bis(hydroxymethyl)tetrahydrofuran, 5-methylfurfural, 5-methylfurfurylalcohol, 5-methyltetrahydrofuran-2-methanol, 2-hexanol, 2,5-dimethyltetrahydrofuran and n-decane were purchased from J&K, which were all analytical grade. H_2_ (>99.99%) and Ar (>99.99%) were supplied by Beijing Analytical Instrument Company. Ultrapure water (resistivity ≥18 MΩ cm) was used in the experiments. All chemicals were used without further purification.

### Synthesis of Nb_2_O_5_ with oxygen-rich vacancy

Nb_2_O_5_ with oxygen-rich vacancy defects (Nb_2_O_5_-Ov) were prepared by the sol-gel method using CTAB as the surfactant. In all, 0.538 g of Niobium(V) oxalate hydrate was dissolved in 20 mL of ultrapure water, and a clear solution was obtained after stirring 2 h at room temperature. In all, 20 mL of CTAB surfactant solution (0.2 M) was added drop wise into the above prepared solution, followed stirring for 2 h at room temperature. Then they were transferred into a dried Teflon autoclave with a capacity of 100 mL at ambient temperature, followed by hydrothermal treatment at 180 °C for 10 h. After being cooled to room temperature, the white precipitate was separated by high-speed centrifugation, washed with ethanol for three times and distilled water for three times, and then dried in an oven at 70 °C for 6 h. The grinded powder was calcined in a tube furnace (Anhui Kemi Machinery Technology Co., Ltd.; Model TFV-1200-50-I-220) at 500 °C for 4 h (reducing gas contains 10 vol.% hydrogen and 90 vol.% argon) to obtain defective Nb_2_O_5_ supports with surface oxygen vacancies.

### Synthesis of M_1_/Nb_2_O_5_-Ov

Pt_1_/Nb_2_O_5_-Ov SAC was synthesized according to the facile adsorption method^39^. In total, 100 mg of Nb_2_O_5_-Ov powders were suspended in 10 mL water. After stirring for 30 min, appropriate amount of H_2_PtCl_6_ solution was added dropwise into the Nb_2_O_5_-Ov dispersion under stirring. After stirring for 4 h and followed by aging for 4 h, the solution was centrifuged and washed with deionized water for several times, and then dried at 60 °C for 12 h. The synthesized catalyst was denoted as Pt_1_/Nb_2_O_5_-Ov. Nb_2_O_5_-Ov supported Pd SAC and Au SAC were prepared by the same method as that for Pt_1_/Nb_2_O_5_-Ov except that H_2_PdCl_4_ and HAuCl_4_ were used to substitute for H_2_PtCl_6_, and the catalyst were denoted as Pd_1_/Nb_2_O_5_-Ov and Au_1_/Nb_2_O_5_-Ov.

### Synthesis of M_n_/Nb_2_O_5_

200 mg of Nb_2_O_5_ powders were suspended in 20 mL water. After stirring for 30 min, appropriate amount of H_2_PtCl_6_ solution was added dropwise into Nb_2_O_5_ dispersion under stirring. Then aqueous solution containing fresh NaBH_4_ was added drop-wise with a continuous magnetic stirring under argon atmosphere, the reaction solution kept on stirring for 2 h to complete the reduction reaction. The obtained granules were collected by centrifuging and washing with ultrapure water for three times (3 × 30 mL) and ethanol twice (2 × 30 mL) and dried in a vacuum oven at 60 °C for 12 h. Nb_2_O_5_ supported Pd nanoparticles and Au nanoparticles were prepared by the same method.

### Characterization

The XRD experiment was performed on Rigaku D/max 2500 with nickel filtered Cu-Kα (λ = 0.154 nm) operated at 40 kV and 20 mA. X-band electron-paramagnetic resonance (EPR) measurement was performed at room temperature using a Bruker EMXplus-9.5/12 EPR spectrometer, with the sample mass of 50 mg. The TEM images of the catalysts were obtained using a JEOL-2100F electron microscope operated at 120 kV. Aberration-corrected high-angle annular dark-field scanning transmission electron microscopy (AC–HAADF–STEM) and element energy dispersive spectroscopy (EDS) mapping images were conducted on a JEOL JEM–ARM200F equipment. The XPS spectra were collected on an ESCA Lab 220i-XL electron spectrometer (VG Scientific) using 300 W Al Kα radiation with a hemispherical energy analyser. The contents of different elements in Pt_1_/Nb_2_O_5_-Ov and Pt_n_/Nb_2_O_5_ catalysts were analyzed by ICP-AES (PROFILE. SPEC, Leeman). The binding energies were calibrated with the C1s level of adventitious carbon at 284.8 eV as the internal standard reference. The XAFS spectra of Pt L3 edge (11564 eV) were collected at 1W1B beamline of Beijing Synchrotron Radiation Facility (BSRF). The beam was tuned by the Si (111) double-crystal monochromators. The energies were calibrated according to the absorption edge of pure Pt foil. The XAFS data were recorded under fluorescence mode by Lytle detector. All collected spectra were processed and analyzed using Athena and Artemis program within the IFEFFIT package. For the XANES analysis, the experimental absorption coefficients as the function of energies were processed by background subtraction and normalization procedure, and reported as “normalized intensity”. Pt foil and PtO_2_ were used as the reference samples. For the extended X-ray absorption fine structure analysis, Fourier transformed data in R space were analyzed by applying metallic Pt model for the Pt-Pt shell. The passive electron factors, S02, were determined by fitting the experimental data of Pt foil and fixing the Pt-Pt coordination number (CN) to be 12, and then fixed for further analysis of the measured samples. The parameters describing the electronic properties (e.g., correction to the photoelectron energy origin, E0) and local structure environment including coordination number (CN), bond distance (R), and Debye Waller factor (σ2) around the absorbing atoms were allowed to vary during the fitting process. The fitted ranges for k and R spaces (*k*^2^ weighted) were *k* = 2.8-10.0 Å^−1^ and *R* = 1.6-3.2 Å. FT-IR spectra of the catalysts with the aldehyde groups and hydroxyl groups absorbed on the catalysts were recorded with a TENSOR 27 spectrometer. The sample (20 mg) of Pt_1_/Nb_2_O_5_-Ov catalyst was dispersed in n-propanal or methanol solution of THF at 160 °C and stirred for 12 h. The suspension was centrifuged and washed using THF to remove the physical absorbed species, and then dried at 100 °C for 12 h. The obtained samples were blended with KBr for IR characterization.

### Hydrogenation reaction

The reaction was carried out in a Teflon-lined stainless steel reactor of 16 mL with a magnetic stirrer. In a typical experiment, suitable amount of reactant, catalyst and solvent were loaded into the reactor. The reactor was sealed and purged with hydrogen to remove the air at room temperature. Then the reactor was placed in a furnace at desired temperature and H_2_ was charged to desired pressure. The stirrer was started with a stirring speed of 600 rpm. After the reaction, the reactor was placed in ice water, the gas was released and a known amount of internal standard (n-decane) was added into the reactor. The liquid reaction mixture in the reactor was transferred into a centrifuge tube. The reactor was washed using THF, which was combined the reaction mixture. The catalyst was separated by centrifugation. The quantitative analysis of the liquid products was conducted using a GC (Agilent 6820) equipped with a flame ionization detector (FID) and a HP-5MS capillary column (0.25 mm in diameter, 30 m in length). Identification of the products and reactant was done using a GC–MS (Agilent 7890B 5977 A, HP-5MS capillary column (0.25 mm in diameter, 30 m in length)) as well as by comparing the retention time with n-decane which was used as the internal standard in GC traces. The conversion of 5-hydroxymethylfurfural and the selectivity of the products were calculated based on the GC data.

### Computational details

Spin-unrestricted density functional theory (DFT) calculations were performed using the Gaussian 16 program package^40^. The free energy of each compound was calculated at the B3LYP/6-311 + G*^41–43^ level, and the entropy were calculated in this work are for a standard state of 413.15 K and 1 atm. The computed Gibbs free energy (G) was obtained by Eq. (1):

1 $$G = E - {\mathrm{TS}} + {\mathrm{ln}}\frac{P}{{P^{\it{0}}}}$$

All spin-polarized DFT calculations in this work were carried out using the Vienna Ab-initio Simulation Package (VASP)^44^. The projector augmented wave (PAW) method^45^ and the Perdew-Burke-Ernzerhof (PBE)^46^ functional utilizing the generalized gradient approximation (GGA)^47^ were applied throughout the calculations. The Brillouin zone integration was performed using 2 × 2 × 1 K-point mesh was used for all these models. The top two layers of all slabs were allowed to fully relax, while the bottom single layer was kept fixed to mimic the bulk region. The kinetic energy cut-off was set as 450 eV, the structure optimization force threshold was 0.03 eV/Å, and the self-consistent calculations applied a convergence energy threshold of 10^−6^ eV. We used a large vacuum height of 15 Å to eliminate the interaction between neighboring slabs. The transition states (TS) of surface reactions were located using a constrained optimization scheme and were verified when (i) all forces on atoms vanish and (ii) the total energy is a maximum along the reaction coordination but a minimum with respect to the rest of the degrees of freedom^48–50^. Vibrational analysis was carried out to ensure that the transition states have only one imaginary frequency along the reaction coordinate (Supplementary Table 6). The adsorption energy of species X on the surface, *E*_ads_(X), was calculated with

2 $$E_{{\mathrm{ads}}}\left( {\mathrm{X}} \right) = E_{{\mathrm{X}}/{\mathrm{slab}}}-E_{{\mathrm{slab}}}-E_{\mathrm{X}}$$

where *E*_X/slab_ is the calculated total energy of the adsorption system, while *E*_slab_ and *E*_X_ are calculated energies of the clean surface and the gas-phase molecule X, respectively. Obviously, a negative *E*_ads_(X) value indicates an exothermic adsorption process. The oxygen vacancy formation energy (*E*_Ov_) was calculated according to

3 $$E_{{\mathrm{Ov}}} = E_{{\mathrm{slab}} - {\mathrm{vac}}} + 1/2E_{{\mathrm{O2}}}-E_{{\mathrm{slab}}}$$

where *E*_slab-vac_ is the total energy of the surface with one oxygen vacancy, and *E*_O2_ is the energy of a gas-phase O_2_ molecule.

## Supplementary information

Supplementary Information

Description of Additional Supplementary Files

Supplementary Data 1

## Data Availability

The primary data that support the plots within this paper and other finding of this study are available from the corresponding author on reasonable request. The DFT structures can be found in the Supplementary Data 1.
