# Supplementary material for: Selective hydrogenation of 5-(hydroxymethyl)furfural to 5-methylfurfural over single atomic metals anchored on Nb2O5
Source: Nat Commun. 2021 Jan 26;12:584. doi: 10.1038/s41467-020-20878-7 (PMC7838200; doi:10.1038/s41467-020-20878-7)
Supplement: Supplementary file 1 — Supplementary Information [file 41467_2020_20878_MOESM1_ESM.pdf]

Supporting Information

Selective Hydrogenation of 5-(Hydroxymethyl)furfural to 5-Methylfurfural over Single Atomic Metals Anchored on Nb_2O_5

Shaopeng Li,[1,2] Minghua Dong,[1,2] Junjuan Yang,[1] Xiaomeng Cheng,[1,2] Xiaojun Shen,[1,2] Shulin Liu,[1,2] Zhi-Qiang Wang[3]*, Xue-Qing Gong[3]*, Huizhen Liu[1,2,4]*, Buxing Han[1,2,4]*

1. Beijing National Laboratory for Molecular Sciences, CAS Key Laboratory of Colloid and Interface and Thermodynamics, Institute of Chemistry, Chinese Academy of Sciences, Beijing 100190, China.

2. School of Chemistry and Chemical Engineering, University of Chinese Academy of Sciences, Beijing 100049, China.

3. Key Laboratory for Advanced Materials, Centre for Computational Chemistry and Research Institute of Industrial Catalysis, School of Chemistry and Molecular Engineering, East China University of Science and Technology, Shanghai 200237, China.

4. Physical Science Laboratory, Huairou National Comprehensive Science Center, Beijing 101407, China.

*Correspondence to: zhiqiangwang@ecust.edu.cn (Zhi-Qiang Wang); xgong@ecust.edu.cn (Xue-Qing Gong); liuhz@iccas.ac.cn (Huizhen Liu); hanbx@iccas.ac.cn (Buxing Han).

Additional figures

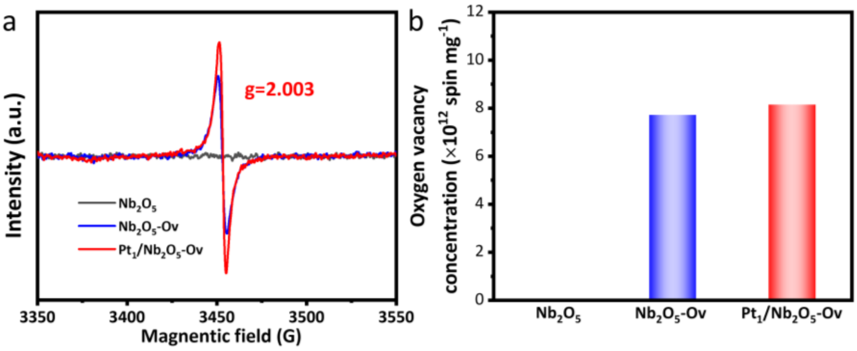

Supplementary Fig. 1 Characterization results from electron-paramagnetic resonance spectra. (a) EPR of Nb_2O_5, Nb_2O_5-Ov and Pt_1/Nb_2O_5-Ov. (b) Quantitative EPR analysis at room temperature. Data for the reduced samples were acquired under vacuum.

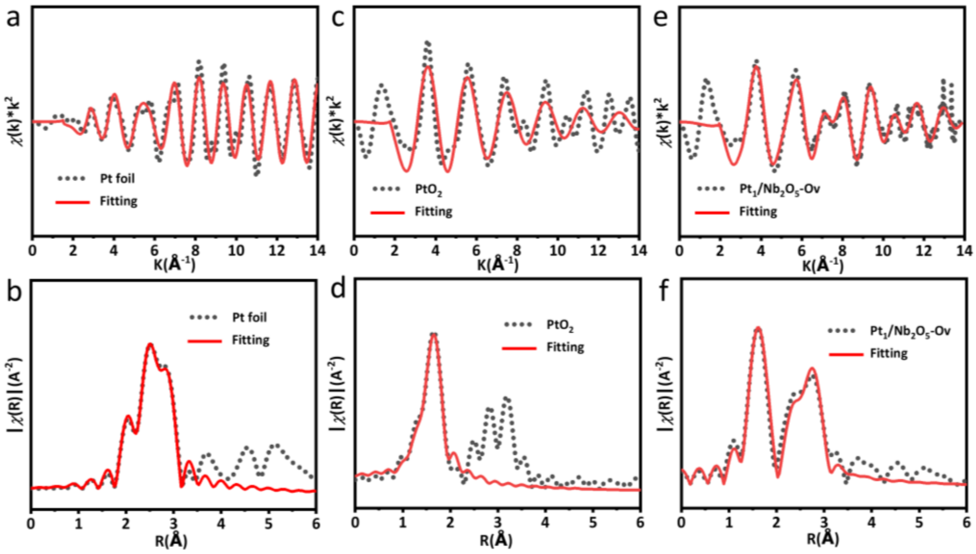

Supplementary Fig. 2 Characterization results from EXAFS. (a, c, e) EXAFS in k space of Pt foil, PtO_2, and Pt_1/Nb_2O_5-Ov. (b, d, f) The corresponding EXAFS fitting of Pt foil, PtO_2, and Pt_1/Nb_2O_5-Ov at R space.

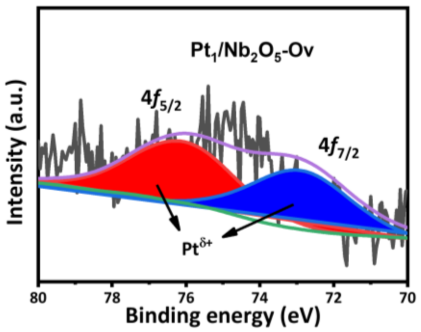

Supplementary Fig. 3 Characterization results from XPS. The Pt 4*f* of Pt_1/Nb_2O_5-Ov.

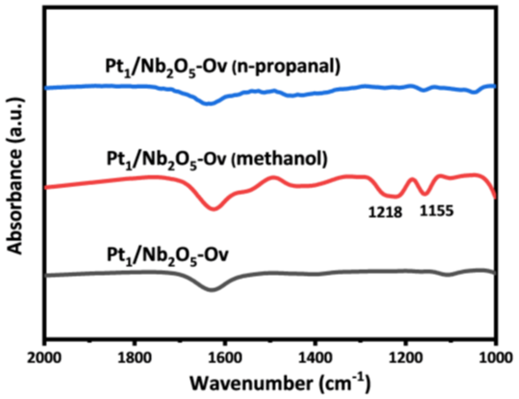

Supplementary Fig. 4 Characterization results from FT-IR. FT-IR spectra of methanol and n-propanal adsorbed on Pt_1/Nb_2O_5-Ov.

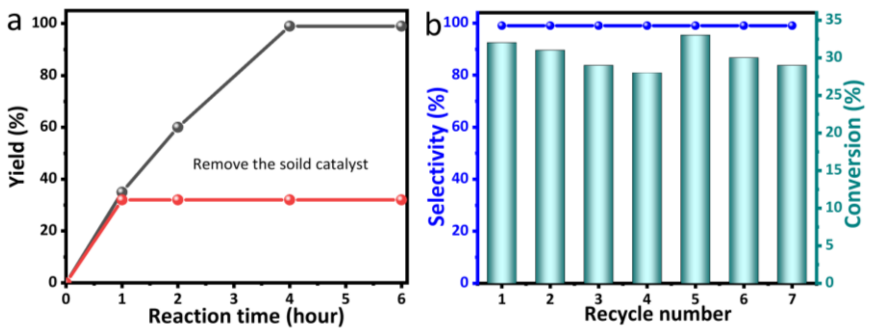

Supplementary Fig. 5 Recycling test of the Pt_1/Nb_2O_5-Ov catalyst. (a) Time-yield plots for MF from selective hydrodeoxygenation of HMF over Pt_1/Nb_2O_5-Ov (black line) or removing Pt_1/Nb_2O_5-Ov after 1 h (red line); (b) Reusability of the Pt_1/Nb_2O_5-Ov. Reaction conditions: HMF (0.3 mmol), catalyst (20 mg), solvent (THF 2 mL), reaction temperature (160°C), H_2 pressure (4.0 MPa), reaction time (1 h), stirring speed (600 rpm).

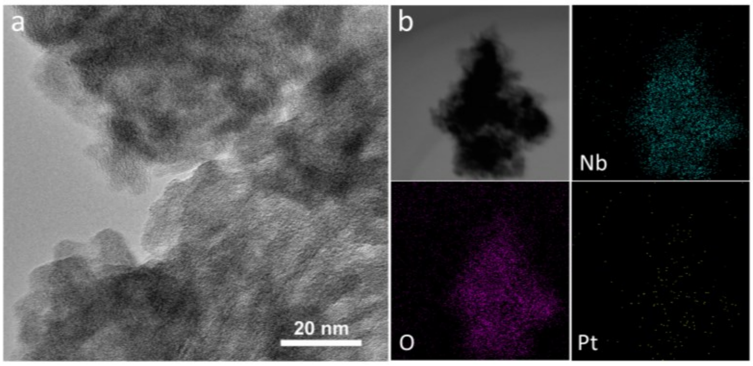

Supplementary Fig. 6 The HR-TEM image and corresponding EDS pattern of Pt_1/Nb_2O_5-Ov. (a) TEM images of the used Pt_1/Nb_2O_5-Ov. (b) EDS mapping images of the used Pt_1/Nb_2O_5-Ov.

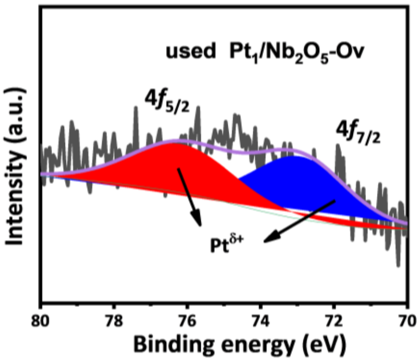

Supplementary Fig. 7 Characterization results from XPS. The Pt 4*f* of the used Pt_1/Nb_2O_5-Ov.

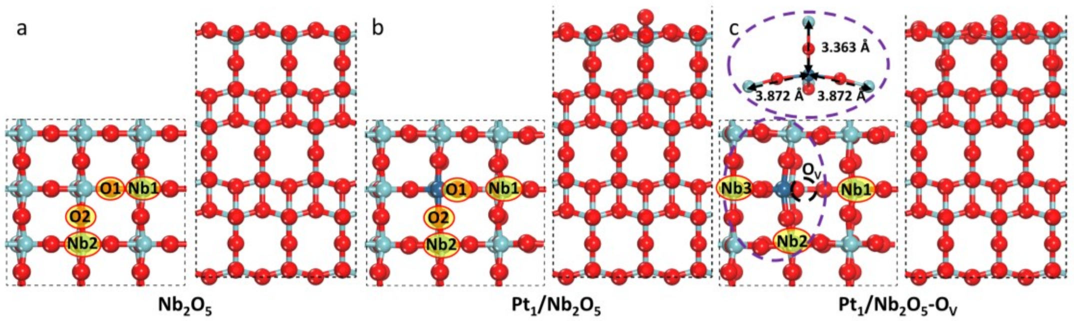

Supplementary Fig. 8 Calculated structures of the stoichiometric. (a) Nb_2O_5, (b) Pt_1/Nb_2O_5, (c) Pt_1/Nb_2O_5-Ov surfaces (Left: top view; Right: side view). Red: O, light blue: Nb, blue: Pt; the dotted circles label the missing oxygens (vacancies). These notations are used throughout the paper.

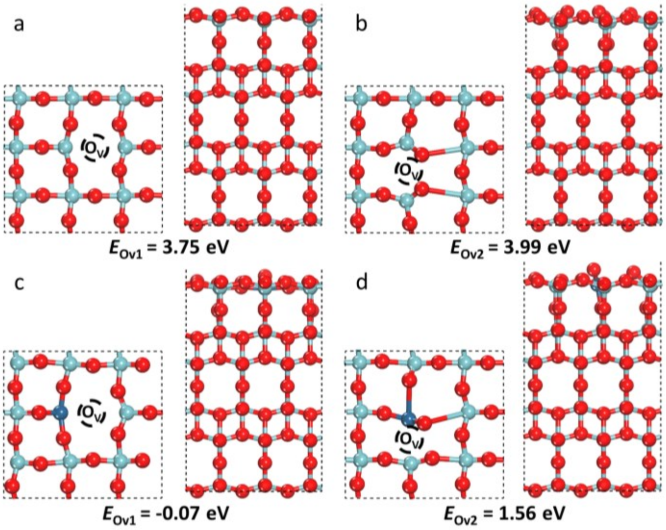

Supplementary Fig. 9 Calculated structures and the oxygen vacancy energies. (a, b) Nb_2O_5 and (c, d) Pt_1/Nb_2O_5 surfaces. The dotted circles label the missing oxygens (vacancies). (Left: top view; Right: side view). Red: O, light blue: Nb, blue: Pt.

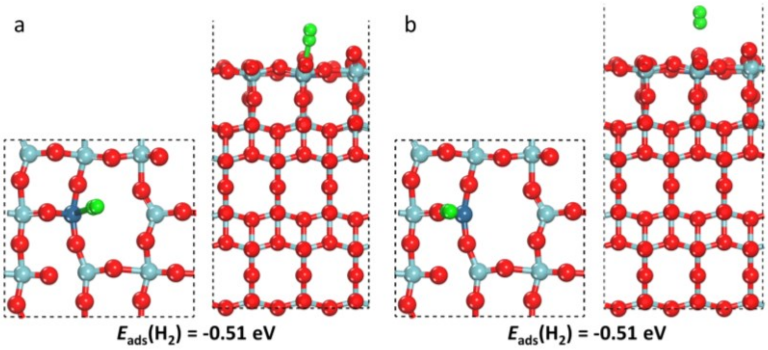

Supplementary Fig. 10 Calculated adsorption energy and structures of H_2 on Pt_1/Nb_2O_5-O_V surfaces. (a) Pt site, (b) O site. (Left: top view; Right: side view). Red: O, light blue: Nb, blue: Pt, green: H.

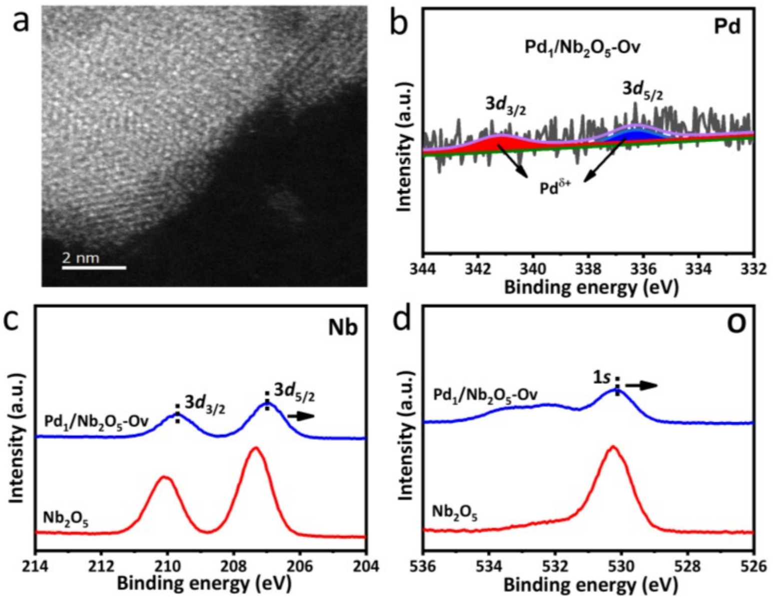

Supplementary Fig. 11 AC–HAADF–STEM image and XPS pattern of Pd_1/Nb_2O_5-Ov. (a) AC–HAADF–STEM image of Pd_1/Nb_2O_5-Ov. The XPS spectra of Pd 3*d* (b), Nb 3*d* (c) and O 1*s* (d) of Nb_2O_5 and Pd_1/Nb_2O_5-Ov.

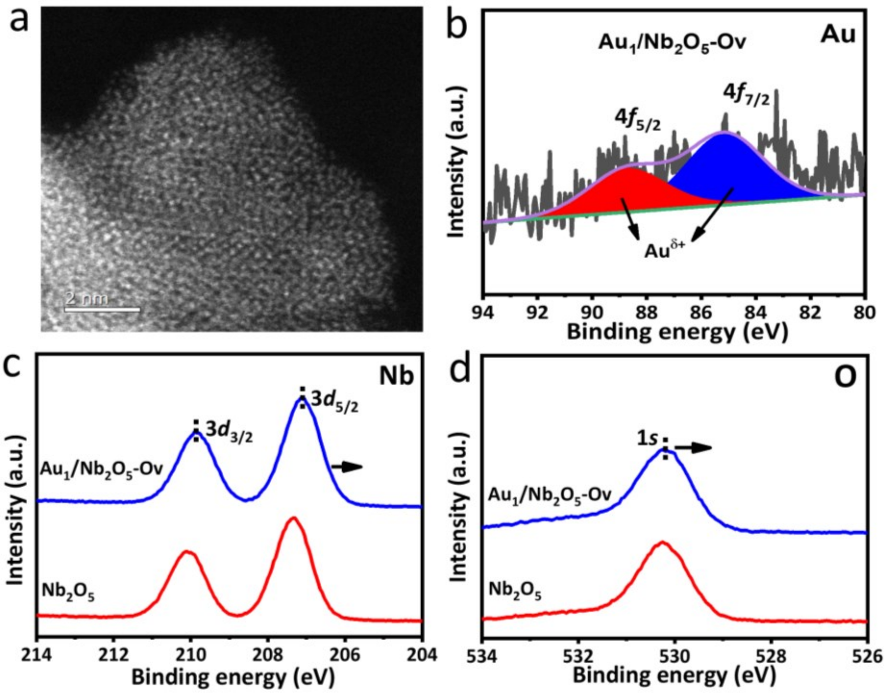

Supplementary Fig. 12 AC–HAADF–STEM image and XPS pattern of Au_1/Nb_2O_5-Ov. (a) AC–HAADF–STEM image of Au_1/Nb_2O_5-Ov. The XPS spectra of Au 4*f* (b), Nb 3*d* (c) and (d) O 1*s* of Nb_2O_5 and Au_1/Nb_2O_5-Ov.

Supplementary Table 1. Structural parameters extracted from quantitative EXAFS curve-fitting.

Sample	Shell	C. N.	$\sigma^2/10^{-3}(Å^2)$	E_0/eV	R/ Å	R-factor
Pt foil	Pt-Pt	12	4.63	8.576	2.76	0.002
PtO_2	Pt-O 1	2	3.10	11.98	1.92	0.017
	Pt-O 2	4	0.87	11.98	2.02	
	Pt-Pt	2	3.80	14.55	3.14	
Pt_1/Nb_2O_5-Ov	Pt-O	2.11	2.93	14.10	2.00	0.019
	Pt-Nb	3.24	5.66	12.15	2.92	

EXAFS fitting details of Pt foil, PtO_2 and Pt_1/Nb_2O_5-Ov, catalyst. The EXAFS data (k-range: -12.5 $Å^{-1}$ and R-range: 1-3.3 Å) were fitted in 1,2 and3 k-weighted R-space. Amplitude reduction factors were evaluated for reference spectra.

Supplementary Table 2. Catalytic Performances of Pt-Based Catalysts for the Conversion of HMF.

Entry	Catalyst	T(°C)	P(MPa)	Product	Conv. (%)	Yield (%)	TOF (h^{-1})	Refrences
1	Pt/C	120	6.2	BHMF	11	2	21.3	1
2	Pt/MCM-41	35	0.8	BHMF	100	98.9	386.3	2
3	Pt_3Sn/SnO_2/rGO	70	2	BHMF	>99	>99	1308.0	3
4	Pt/AC	120	3	DMF	67.4	32.6	33.7	4
5	Pt/rGO	120	2	DMF	100	73.2	50.0	4
6	Pt/NaY	220	1.5	DMF	100	30.4	195.1	5
7	PtCo@HCS[a]	180	2	DMF	100	98	32.5	6
8	Pt_1/Nb_2O_5-Ov	160	4	MF	>99	>99	1875.3	This work

[a]HCS, hollow carbon spheres.

Supplementary Table 3. Calculated the entropy and energy of each compound at the 413.15 K.

	H_2O	H_2	HMF	MF	DMF
S/cal/mol/K	45.08	31.13	90.51	84.18	81.81
E/eV	-14.22	-88.7	-6.76	-94.95	-89.97

Supplementary Table 4. The hydrodeoxygenation of MF to DMF over Pt_1/Nb_2O_5-Ov.

Entry	Catalyst	Temperature (°C)	Time (h)	Conversion (%)	Yield (%)
1	Pt_1/Nb_2O_5-Ov	160	2	trace	-

Reaction conditions: MF (0.3 mmol), catalyst (20 mg), solvent (THF 2 mL), H_2 pressure (4 MPa), stirring speed (600 rpm).

Supplementary Table 5. Hydrodeoxygenation of different substrates over the Pt_1/Nb_2O_5-Ov catalyst[a].

Entry	Substrate	Temperature / °C	Time / h	Product	Conversion / %	Yield. / %
1	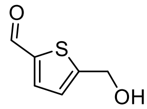	160	4	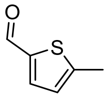	>99	98
2	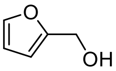	160	4	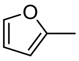	>99	>99
3	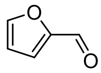	160	4	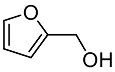	trace	trace
4	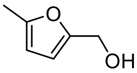	160	4	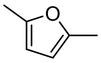	>99	>99
5	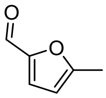	160	4	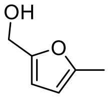	trace	-
6	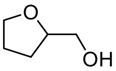	160	6	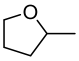	trace	-
7[b]	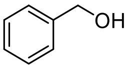	160	1	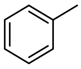	24	24
8[b]	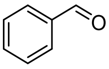	160	1	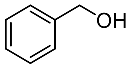	55	45
9[b]	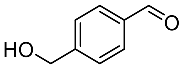	160	1	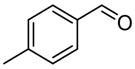	>99	23
10[c]	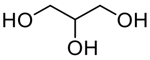	200	6	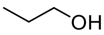	11	9
11[c]	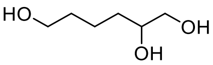	200	6	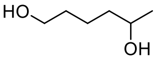<br>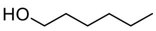	26	14.6<br>11.4
12[b]	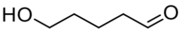	160	2	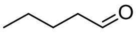	trace	trace
13[b]	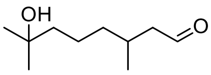	160	4	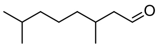	trace	trace

[a] Reaction conditions: substrate (0.3 mmol), catalyst (20 mg), solvent (THF 2 mL), H_2 pressure (2.0 MPa), stirring speed (600 rpm). [b] solvent (ethanol 2 mL). [c] 10wt% solution (2 mL).

Supplementary Table 6. The imaginary frequency of TS.

Structure	Frequency (cm^{-1})
TS1	971.15
TS2	319.98

Fractional coordinates for optimized DFT structures

Fractional coordinates are provided in CIF-format and are labeled according to the nomenclature introduced in Supplementary Fig. 8c. The structures with CIF-format can be found in the Supplementary Data 1.

AutoCreatByScript: O Nb Pt

1.00000000000000

12.2555099999999992	0.0000000000000000	0.0000000000000000
0.0000000000000000	11.4381599999999999	0.0000000000000000
0.0000000000000000	0.0000000000000000	32.7415599999999998

a=12.25551; b=11.43816; c=32.74156

alpha=90.00; beta=90.00; gamma=90.00

O	Nb	Pt
134	53	1

Selective dynamics

Direct

0.1046750000000003	0.1250980000000013	0.0005060000000014
0.4380080000000035	0.1250980000000013	0.0005060000000014
0.7713419999999971	0.1250980000000013	0.0005060000000014
0.1046750000000003	0.4584309999999974	0.0005060000000014
0.4380080000000035	0.4584309999999974	0.0005060000000014
0.7713419999999971	0.4584309999999974	0.0005060000000014
0.1046750000000003	0.7917649999999981	0.0005060000000014
0.4380080000000035	0.7917649999999981	0.0005060000000014
0.7713419999999971	0.7917649999999981	0.0005060000000014
0.2690119999999965	0.2918859999999981	0.0153400000000019
0.6023449999999997	0.2918859999999981	0.0153400000000019
0.9356790000000004	0.2918859999999981	0.0153400000000019
0.2690119999999965	0.6252190000000013	0.0153400000000019
0.6023449999999997	0.6252190000000013	0.0153400000000019
0.9356790000000004	0.6252190000000013	0.0153400000000019
0.2690119999999965	0.9585530000000020	0.0153400000000019
0.6023449999999997	0.9585530000000020	0.0153400000000019
0.9356790000000004	0.9585530000000020	0.0153400000000019
0.1019549999999967	0.2917440000000013	0.0736300000000014
0.4352879999999999	0.2917440000000013	0.0736300000000014
0.7686210000000031	0.2917440000000013	0.0736300000000014
0.1019549999999967	0.6250769999999974	0.0736300000000014
0.4352879999999999	0.6250769999999974	0.0736300000000014
0.7686210000000031	0.6250769999999974	0.0736300000000014
0.1019549999999967	0.9584100000000007	0.0736300000000014
0.4352879999999999	0.9584100000000007	0.0736300000000014
0.7686210000000031	0.9584100000000007	0.0736300000000014

0.2682059999999993	0.2919070000000019	0.1319280000000020
0.6015390000000025	0.2919070000000019	0.1319280000000020
0.9348719999999986	0.2919070000000019	0.1319280000000020
0.2682059999999993	0.6252410000000026	0.1319280000000020
0.6015390000000025	0.6252410000000026	0.1319280000000020
0.9348719999999986	0.6252410000000026	0.1319280000000020
0.2682059999999993	0.9585739999999987	0.1319280000000020
0.6015390000000025	0.9585739999999987	0.1319280000000020
0.9348719999999986	0.9585739999999987	0.1319280000000020
0.0992460000000008	0.1250860000000031	0.1467620000000025
0.4325800000000015	0.1250860000000031	0.1467620000000025
0.7659129999999976	0.1250860000000031	0.1467620000000025
0.0992460000000008	0.4584189999999992	0.1467620000000025
0.4325800000000015	0.4584189999999992	0.1467620000000025
0.7659129999999976	0.4584189999999992	0.1467620000000025
0.0992460000000008	0.7917520000000025	0.1467620000000025
0.4325800000000015	0.7917520000000025	0.1467620000000025
0.7659129999999976	0.7917520000000025	0.1467620000000025
0.0967384831806638	0.2919264668021148	0.1984400293401334
0.7633702939764444	0.2918463737024006	0.1982576030819374
0.0971206506621761	0.6250909529761465	0.1983878578118828
0.7634294359203646	0.6250966884819221	0.1982460873406816
0.0966932167884315	0.9582605589000740	0.1984300736910830
0.7633422942710378	0.9583447604589306	0.1982478609843255
0.4297374153903777	0.2919142787076252	0.1983488579934903
0.4298853475262861	0.6250941586259811	0.1983060467367977
0.4297712531649505	0.9582517621830781	0.1983427581346141
0.9279896457040449	0.1250987434243782	0.2131721172285505
0.5949145351940190	0.4584154001893054	0.2134919369321027
0.9287486032954083	0.4585397009892780	0.2136394736799698
0.5949168655922191	0.7918382706848894	0.2134986203349254
0.2604870360221487	0.1250587220288560	0.2131724963624038
0.5947379109047489	0.1251159969797582	0.2132821556504050
0.9287548278316127	0.7916380197975730	0.2136479386291137
0.2614669053601289	0.4583511239489104	0.2136241332619642
0.2614724407113575	0.7918740188883564	0.2136203968619255
0.0953750192939599	0.1250693949496435	0.2715405390907309
0.0971147257918388	0.4596204952264579	0.2718951093591232
0.7634517416280708	0.4585604165740847	0.2713297929615547
0.7634646167677192	0.7915705068191381	0.2713295617947069
0.0971304490535625	0.7904640380633817	0.2718950335460938
0.7632650214719167	0.1250583530656517	0.2713001901858339
0.4287934200799147	0.1250510725788244	0.2710733326445434
0.4296972187371549	0.4603203353797182	0.2717287006656023

0.4296637932954322	0.7897674883628688	0.2717353462084565
0.9319189361880247	0.4585762443060655	0.3295517284746268
0.9319165758150081	0.7915576413163434	0.3295637208441026
0.9308058116940464	0.1250439463269523	0.3294885750261653
0.2635185682498307	0.1250808948761115	0.3291619754539143
0.2646192950144067	0.7911650629360718	0.3300675558900302
0.5980910335082473	0.1250352245843126	0.3290300291172414
0.2646234202365119	0.4589115597625991	0.3300530164015094
0.5989311299406582	0.4594414149297243	0.3295998084379418
0.5989502571743724	0.7906487414441394	0.3295896953984611
0.0984038125335431	0.6250457919591238	0.3467621836557412
0.7653917003672691	0.6250401390263491	0.3449407998099518
0.0961943523013620	0.2922147753276315	0.3451519070453558
0.0962023216426950	0.9578989733830036	0.3451426884650150
0.7629578203353753	0.2915987060124003	0.3444569939178252
0.7629555862741287	0.9584858186792228	0.3444588727456003
0.4305134026630895	0.6250478559657293	0.3455502428024928
0.4290752554803171	0.2925120667009361	0.3440310428832525
0.4290599888767632	0.9575799936111200	0.3440272752333387
0.4246901410599921	0.1250470160755480	0.3940376652039960
0.0952249583435874	0.7916932883949042	0.3974546254629856
0.0952886426134878	0.4584130692123413	0.3974585186373547
0.7617019902256740	0.4584301540717542	0.3955905980373277
0.7616804484742511	0.7916455246705656	0.3955893843150073
0.0921254821265388	0.1250492868105925	0.3960578745257074
0.7598696712238603	0.1250392432599752	0.3964994427224630
0.4285716792412718	0.7922050860278211	0.3968042906576499
0.4285356387150160	0.4578874466522357	0.3967970915090651
0.9324303803556799	0.6250174527108168	0.4133414922763171
0.9241819980998438	0.2920988843618146	0.4111505904056120
0.9241796000060032	0.9579773813058946	0.4111547693554463
0.2636559533761375	0.6250408339289625	0.4159515500451852
0.5914775448173848	0.2913170631876459	0.4095032404490519
0.5914643946360106	0.9587851749490137	0.4095004755538910
0.2564817037160334	0.2918066407754727	0.4121595503737812
0.2564717510037164	0.9582458408300001	0.4121553641068004
0.5966481006185710	0.6250306095885789	0.4129600395285075
0.0997555617931737	0.6250564508808697	0.4730802251252829
0.7725003651296061	0.6250583609492170	0.4684890110233368
0.4292371055069948	0.2788086520199484	0.4676184917585288
0.4292252938172117	0.9714850192542571	0.4676097684462406
0.7522710481620022	0.3005063555074715	0.4694829809991091
0.7522512579070352	0.9495712541602809	0.4694792091614182
0.0848564676180657	0.2854729613920871	0.4702366964737550

0.0848752556488260	0.9647111163755776	0.4702371578426683
0.4393291162343874	0.6250697596549193	0.4714142702747652
0.2314791599114371	0.6249246385393541	0.5563379522861980
0.9322118840212770	0.6250762303095226	0.5414169326501056
0.2397825289283950	0.2769578301455380	0.5352191987249559
0.2397961798760778	0.9731678054555774	0.5352291992225030
0.5728510430959460	0.3043965376899503	0.5389456559204148
0.5728736782126918	0.9457552098110376	0.5389223153526921
0.3862237883151764	0.4502418319895083	0.5277218207415386
0.3862684189367631	0.7999890579058885	0.5276946388191776
0.7290064651582545	0.1250684502870615	0.5337912477647183
0.8857050551941681	0.3001860051783070	0.5411788035341618
0.8857903806801317	0.9499920046897491	0.5411324548705234
0.7309232154813737	0.7708008762808352	0.5403624398863778
0.7309131894517608	0.4794444045610722	0.5403565127446199
0.0602818576864900	0.1250415846896321	0.5432595443091922
0.0794896902960987	0.4526347691003026	0.5389850883974350
0.0795656213127501	0.7974344343726947	0.5389865014831875
0.4131966938617996	0.1250790315110344	0.5396000313261838
0.4137089999999972	0.2918140000000022	0.0158879999999968
0.7470420000000004	0.2918140000000022	0.0158879999999968
0.0803749999999965	0.2918140000000022	0.0158879999999968
0.4137089999999972	0.6251469999999983	0.0158879999999968
0.7470420000000004	0.6251469999999983	0.0158879999999968
0.0803749999999965	0.6251469999999983	0.0158879999999968
0.4137089999999972	0.9584809999999990	0.0158879999999968
0.7470420000000004	0.9584809999999990	0.0158879999999968
0.0803749999999965	0.9584809999999990	0.0158879999999968
0.1235250000000008	0.2918209999999988	0.1313790000000026
0.4568579999999969	0.2918209999999988	0.1313790000000026
0.7901909999999930	0.2918209999999988	0.1313790000000026
0.1235250000000008	0.6251540000000020	0.1313790000000026
0.4568579999999969	0.6251540000000020	0.1313790000000026
0.7901909999999930	0.6251540000000020	0.1313790000000026
0.1235250000000008	0.9584880000000027	0.1313790000000026
0.4568579999999969	0.9584880000000027	0.1313790000000026
0.7901909999999930	0.9584880000000027	0.1313790000000026
0.7395591494270229	0.7917615370150367	0.2135775210643072
0.7393193094367616	0.1250971830347171	0.2135326479956673
0.0727085108702307	0.1250961388978760	0.2137435493781770
0.7395545254852981	0.4584330543074255	0.2135765957587169
0.0734816091941921	0.4585314083764631	0.2139952591726909
0.0734866378569975	0.7916553095893017	0.2139942706924708
0.4052592422640552	0.1250766376594291	0.2135549136591275

0.4062455735296422	0.4585776163116938	0.2140753199105416
0.4062570249189277	0.7915921156634166	0.2140775250399568
0.1200484072910713	0.4586358115636681	0.3295674323315809
0.7870506836990887	0.4579818790290721	0.3288309346962276
0.7870507137009517	0.7921077287478379	0.3288330729734987
0.1200470128132743	0.7914645541610899	0.3295678121372028
0.1189888664478994	0.1250540466493206	0.3291913379338003
0.7860121966917453	0.1250425281332521	0.3288268172213810
0.4532477832332356	0.1250478899739614	0.3287969204144091
0.4539675700861148	0.7901252491618805	0.3296027371488895
0.4539495625044627	0.4599646772188165	0.3296005717843108
0.0769208367745501	0.6250460580232340	0.4134048907518539
0.7411872834698831	0.6250360301255641	0.4110987028679308
0.7356103076924343	0.2921007867769403	0.4112570435984067
0.7355956825001237	0.9579842241330444	0.4112528134674037
0.0690161888471004	0.9588544391867516	0.4120592244861557
0.0690198101829151	0.2912489741854610	0.4120575271073536
0.4016537683121231	0.2910915181319456	0.4108897864050834
0.4016461487872588	0.9590088481320332	0.4108859125119871
0.4095520558188090	0.6250456753202298	0.4151885685610323
0.0926835913737988	0.6250698141443869	0.5300641591856180
0.7866195387748985	0.6250999974300260	0.5258380981222645
0.4266710020462290	0.9575244020455012	0.5253330434976995
0.4266215587991636	0.2928084395724801	0.5253414101832371
0.7455374675494134	0.2941225059012527	0.5270402579101339
0.7455855954516731	0.9560020376456498	0.5270320813265934
0.0961956434334530	0.2871868952673686	0.5277857786230936
0.0962110145530493	0.9629538965037145	0.5277860229205410
0.3667232892220451	0.6251986412025610	0.5245968267100587

Supplementary References

1. Chidambaram, M. & Bell, A. T. A two-step approach for the catalytic conversion of glucose to 2,5-dimethylfuran in ionic liquids. *Green Chem.* **12**, 1253−1262 (2010).
2. Chatterjee, M., Ishizaka, T. & Kawanami, H. Selective hydrogenation of 5-hydroxymethylfurfural to 2,5-bis-(hydroxymethyl)furan using Pt/MCM-41 in an aqueous medium: a simple approach. *Green Chem.* **16,** 4734−4739 (2014).
3. Shi, J., Zhang, M., Du, W., Ning, W. & Hou, Z. SnO_2-isolated Pt_3Sn alloy on reduced graphene oxide: an efficient catalyst for selective hydrogenation of CO in unsaturated aldehydes. *Catal. Sci. Technol.* **5,** 3108−3112 (2015).
4. Shi, J., Wang, Y., Yu, X., Du, W. & Hou, Z. Production of 2,5-dimethylfuran from 5-hydroxymethylfurfural over reduced graphene oxides supported Pt catalyst under mild conditions. *Fuel* **163**, 74−79 (2016).
5. Nagpure, A. S., Lucas, N. & Chilukuri, S. V. Efficient preparation of liquid fuel 2,5-dimethylfuran from biomass-derived 5-hydroxymethylfurfural over Ru-NaY catalyst. *ACS Sustainable Chem. Eng.* **3,** 2909−2916 (2015).
6. Wang, G. H. et al. Platinum-cobalt bimetallic nanoparticles in hollow carbon nanospheres for hydrogenolysis of 5-hydroxymethylfurfural. *Nat. Mater.* **13,** 293−300 (2014).
