# Supplementary material for: Selective hydrogenation of 5-(hydroxymethyl)furfural to 5-methylfurfural over single atomic metals anchored on Nb2O5
Source: Nat Commun. 2021 Jan 26;12:584. doi: 10.1038/s41467-020-20878-7 (PMC7838200; doi:10.1038/s41467-020-20878-7)
Supplement: Supplementary file 2 — Description of Additional Supplementary Files [file 41467_2020_20878_MOESM2_ESM.docx]

Description of Additional Supplementary Files

File Name: Supplementary Data 1

Description: The structures with CIF-format files of Fig. 5a-5f, Fig. 6b-6i, Supplementary Fig. 8a-8c, Supplementary Fig. 9a-9d and Supplementary Fig. 10a-10b.
